# Skin tissue regeneration for burn injury

**DOI:** 10.1186/s13287-019-1203-3

**Published:** 2019-03-15

**Authors:** Anastasia Shpichka, Denis Butnaru, Evgeny A. Bezrukov, Roman B. Sukhanov, Anthony Atala, Vitaliy Burdukovskii, Yuanyuan Zhang, Peter Timashev

**Affiliations:** 1 Institute for Regenerative Medicine, Sechenov University, Moscow, Russia; 2 Sechenov Biomedical Science and Technology Park, Sechenov University, Moscow, Russia; 3 Department of Urology, Sechenov University, Moscow, Russia; 4 Wake Forest Institute for Regenerative Medicine, Wake Forest School of Medicine, Winston-Salem, NC USA; 5 Baikal Institute of Nature Management, Siberian Branch of the Russian Academy of Sciences, Ulan-Ude, Russia; 6 Research Center “Crystallography and Photonics” RAS, Institute of Photonic Technologies, Troitsk, Moscow, Russia; 7 Departments of Polymers and Composites, N.N. Semenov Institute of Chemical Physics, Moscow, Russia

**Keywords:** Burns, Skin regeneration, Cell-based therapy, Stem cells, Skin substitutes

## Abstract

The skin is the largest organ of the body, which meets the environment most directly. Thus, the skin is vulnerable to various damages, particularly burn injury. Skin wound healing is a serious interaction between cell types, cytokines, mediators, the neurovascular system, and matrix remodeling. Tissue regeneration technology remarkably enhances skin repair via re-epidermalization, epidermal-stromal cell interactions, angiogenesis, and inhabitation of hypertrophic scars and keloids. The success rates of skin healing for burn injuries have significantly increased with the use of various skin substitutes. In this review, we discuss skin replacement with cells, growth factors, scaffolds, or cell-seeded scaffolds for skin tissue reconstruction and also compare the high efficacy and cost-effectiveness of each therapy. We describe the essentials, achievements, and challenges of cell-based therapy in reducing scar formation and improving burn injury treatment.

## Introduction

Burns remain as one of the most common injuries worldwide, with more than one million patients annually in the USA alone [1]. A burn ensues after the skin is damaged by heat, radiation, electricity, or chemicals. Serious complications of deep or widespread burns can happen, e.g., sepsis due to bacterial infection, shock caused by hypovolemia, or scaring tissue contraction after improper wound healing. The skin damage causes the death of skin cells, leading to an enormous loss of body fluids that is followed by dehydration, electrolyte imbalance, and renal and circulatory failure. Another serious threat to lives of burn patients is an infection. The burned skin is extremely susceptible to bacteria and other pathogens, due to the loss of protection by intact layers of the skin. Each of these complications can be fatal or make a patient suffer. Therefore, it is critical to promptly cover a burn injury using an appropriate approach to prevent them and save patients’ lives, besides providing intravenously fluids and nutrients to offset dehydration and replace lost proteins.

The survival rates of patients with burns have significantly improved due to the application of various skin grafts over the last decades. Despite wide use, autologous skin grafts are deficient in the treatment of severe burns for patients with limited donor site area [2, 3]. Skin substitutes, especially cell-based ones, play critical role in overcoming this scarcity. The cumulative effect of cell-sheets, scaffolds, cell-scaffolds, and hydrogels with healing promoting factors triggers, accelerates, and enhances wound healing and re-epithelialization that leads to a reduction in scar formation and prevention of burn injury complication. Skin substitutes have shown high efficacy and cost-effectiveness compared to autologous skin replacement [12, 13]. In this study, we focus on discussing the essentials, achievements, and challenges of cell-based therapy for skin tissue regeneration in the treatment of burn injury.

The skin plays an important role which cannot be overestimated; its functioning ensures homeostasis and protects us from aggressive and causative agents in the environment. It is constantly involved in numerous processes: water balance and temperature regulation, signal perception, hormone, neuropeptide and cytokine production and activation, etc. [4]. The skin is formed by three main layers (the epidermis, the dermis, and the hypodermis) with its appendages (hair, sweat and sebaceous glands, sensory neurons, blood and lymph vessels, etc.) [5]. The entire skin tissue contains various cells (epidermal, stromal, endothelial, and neuronal cells) and the extracellular matrix (ECM). Cells, growth factors, and matrix are the basic elements for use in the skin regeneration and replacement after an injury.

## Skin anatomy

The skin is a complex tissue, and its structure is presented by the epidermis, the dermis, the hypodermis, and skin appendages [5].

The external first layer—the epidermis—is the main barrier between the environment and internal organs and tissues. It is structured in layers (strata): horny layer (stratum corneum), clear layer (stratum lucidum), granular layer (stratum granulosum), spinous layer (stratum spinosum), and basal layer (stratum basale) [6, 7]. The epidermis is thin and stratified and consists of cell populations such as keratinocytes, Merkel cells, melanocytes, and Langerhans cells [8]. Keratinocytes are the major cell component of the epidermis and responsible for its stratified structure; they form numerous and tight intercellular junctions. Melanocytes, which synthesize melanin (pigment absorbing UV radiation and protecting from its negative effects), are located in the basal layer (stratum basale) and form dendrites that can reach the spinous layer (stratum spinosum) [7]. Merkel cells, which are responsible for the mechanic perception, are also found in the stratum basale (above the basement membrane). Langerhans cells are distributed in the stratum spinosum and involved in immune protection: they act as an antigen-presenting cell and engulf pathogens or other foreign matter [5].

Being the main cell component in all epidermal layers, keratinocytes ensure keratinization due to their differentiation starting in the basal layer [9]. While differentiating and migrating towards a skin surface, keratinocytes become anucleated and have clustered keratin in the stratum granulosum. Then they flatten and die in the stratum corneum. Corneocytes (differentiated keratinocytes) have tight intercellular junctions that prevent water evaporation and skin dehydration, but they are expulsed because of the desmosome loss [10]. This process is involved in desquamation (i.e., skin peeling). However, the epidermis has no direct blood supply, and delivery of nutrients and elimination of residuals occur due to the diffusion from the underlying dermis through the epidermal basement membrane [8, 11]. The basement membrane is a semipermeable layer which is formed by ECM components such as collagen type IV, nidogen, laminin, and perlecan [12, 13].

Beneath the epidermis, there is the dermis which forms a thick layer mainly consisting of the connective tissue and ECM [7, 8]. It is more heterogeneous than the epidermis, and different structures like blood and lymph vessels, sweat and sebaceous glands, and hair follicles are located there. It can be divided into two layers: papillary and reticular. The first one is thin and superficial and presented by the flowing connective tissue, which includes reticular, elastic, and non-organized collagen (mostly type III) fibers and capillaries. The latter one is thick and deep and presented by the compact connective tissue, which has crosslinked elastic and well-organized collagen (type I and III) fibers and large blood vessels [7]. The connective tissue mainly consists of collagen, which enables the skin’s strength, but there are also elastin (elasticity and flexibility) and proteoglycans (hydration and viscosity) [12]. It is constantly remodeled because of the action of proteolytic enzymes (matrix metalloproteinases) synthesized by fibroblasts, neutrophils, keratinocytes, etc., and involved in numerous processes in the skin [14, 15]. The main cell type of the dermis is fibroblasts, which produce components of the ECM (collagen, elastin, and proteoglycans) and secrete various growth factors (TGF-β), cytokines (TNF-α), and matrix metalloproteinases. This “cocktail” ensures the formation of the ECM and keratinocyte proliferation and differentiation [16]. Therefore, fibroblasts are essential for the skin remodeling and wound healing [17]. Moreover, various immune cells (e.g., dendritic cells, leukocytes) are found and can migrate through the dermis [4].

Between the dermis and muscles, the hypodermis (subcutaneous tissue) is located [7]. It protects the internal tissues and organs from cold and trauma, provides energy, and participates in the hormone synthesis (e.g., estrone, leptin) [4]. The hypodermis is formed by adipocytes structured in lobules. These lobules are separated with the septa from the connective tissue and contain nerves and lymphatic and microvascular network, which ensures nutrient and oxygen delivery [7].

Moreover, the skin structure also includes the skin appendages [6, 18], e.g., nails, hair follicles, sweat glands, and sebaceous glands. Hair follicles, which are distributed all around the body (except palms and soles), are formed by basal cells in the basement membrane and responsible for the body temperature control and mechanic perception [6]. Keratinized and dead cells compose nails [7]. At the base of the hair follicles, there are sebaceous glands which produce sebum (oily substance), which ensures the skin and hair lubrication and waterproofness [19]. Sweat glands secrete sweat onto a skin surface [20], and ceruminous and mammary glands are the changed sweat glands that are responsible for the cerumen and milk (respectively) production [7, 21].

The recent findings have shown that the skin has its own stem cells which are rather heterogeneous and can be divided into various subtypes: epidermal, follicular, hematopoietic, melanocyte and sebaceous gland stem, mesenchymal stem-like, and neuronal progenitor cells [6, 22].

## Skin tissue regeneration processes

Skin would healing is a systematic process, traditionally including four overlapping classic phases [23, 24]: hemostasis (coagulation), inflammation (mononuclear cell infiltration), proliferation (epithelialization, fibroplasia, angiogenesis, and formation of granulation tissue), and maturation (collagen deposit or scaring tissue formation). Several factors influence skin healing after burn injuries, e.g., the causes, the degree and size of burn, and the patient’s general condition and types of the graft or materials for covering burn wounds.

Depending on burn severity, the healing process may result in different consequences. Superficial burns recover within two weeks and cause minimal scarring. The re-epithelization of partial thickness burns is ensured by keratinocyte migration from skin dermal appendages within a few hours of the injury. In deeper burns, the healing starts around the edges, but not at the center because of the necessity of rapid wound closure [25–27]. The acceleration of early cell proliferation ensuring the rapid burn healing occurs due to dendritic cells releasing various factors. So, agents enhancing dendritic cells are considered as therapeutics improving burn wound care [28]. Angiogenesis during burn healing is induced by hypoxia-inducible factor 1 [29] and angiogenic cytokines such as VEGF and CXCL12 [30] and ensured by the increase in endothelial progenitor cell blood level correlating with the skin area burnt [30, 31]. The increased contraction is ensured by the activation of the TGF-β pathway that causes remodeling and scar formation [26].

Compared to other wound types, burns may have systemic effects [32, 33], influencing almost all body systems and causing changes in lung, kidney, heart, liver, gastrointestinal tract, bone marrow, and lymphoid organ functioning and multiple organ dysfunction syndrome [32]. At the burn site, inflammatory mediators such as tumor necrosis factor alpha (TNF-α) and interleukins 6, 8, and 1-beta, responsible for systemic effects, are released. Their concentration in serum correlates with the burn surface area. The rise in their concentrations is considered to increase risk of infections, multiple organ dysfunction syndrome, and death [34–36].

Moreover, burn healing is followed by significant immune imbalance [32]. At an early stage, the suppression of bone marrow leads to lymphoid and myeloid immune cell dysfunction, which makes infections resistant to common therapy, and may even cause sepsis. These infectious complications result in wound healing delay [1, 37]. Neutrophils are shown to overexpress heat shock proteins, leading to an increase in oxidative activity and a decrease in apoptosis. Thus, the inflammation phase is prolonged, and the wound site overexposed with growth factors and inflammatory mediators [37–39].

## Cell types used in skin regeneration

Cells are the main component of the tissue-engineered skin used for burn therapies (Table 1)**.** They include both stem and somatic cells and can be divided into three main groups: autologous, allogeneic, and xenogeneic. One of the main trends in choosing a cell type for patient treatment is the use of autologous cells as they do not cause immune rejection and their tumorigenicity is low due to the absence of epigenetic manipulations. Nowadays, animal cells are not widely used for skin tissue regeneration, only ECM or its components that they synthesize. Plant stem cells, which are commonly applied in cosmetics, can be interesting as they have no use limitations when compared to animal and human cells. Of course, they cannot be used in skin substitute development as a cell component; but they can provide bioactive substances, which can improve the wound healing process [40].

**Table 1:** Somatic and stem cells used in skin tissue regeneration

Cell types (Refs.)	Origin	Source	CT	Examples of commercial products and their indications
Fibroblasts [49–57]	Allogeneic	Skin	Yes	Apligraft - Venous leg ulcers - Diabetic foot ulcers OrCel - Partial-thickness burns
		Neonatal foreskin	Yes	TransCyte - Full-thickness and deep partial-thickness burns - Partial-thickness burns Dermagraft - Full-thickness diabetic foot ulcers
		Fetus	Yes	ND
	Autologous	Skin	Yes	TissueTech Autograft System - -Diabetic foot ulcers Hyalograft 3D - Diabetic ulcer - Cartilage engineering
Keratinocytes [55, 56, 58–64]	Allogeneic	Skin	Yes	Apligraft - Venous leg ulcers - Diabetic foot ulcers OrCel - Partial-thickness burns
		Neonatal foreskin	Yes	Lyphoderm - Chronic venous ulcer - Partial-thickness burns
		Fetus	Yes	ND
	Autologous	Skin	Yes	Epicel - Deep dermal or full thickness burns TissueTech Autograft System - Diabetic foot ulcers Bioseed-S - Chronic venous leg ulcers CellSpray - Partial and deep partial-thickness burns Karocells - Partial and deep partial-thickness burns
		Outer root sheath of scalp hair follicles	Yes	EpiDex - Recalcitrant vascular leg ulcers - Partial-thickness burns
ESC [65]	Autologous	Epidermis (basal layer)	Yes	ND
MMSC [66–71]	Allogeneic	Adipose tissue	Yes	ND
		Umbilical cord	Yes	ND
		Bone marrow	Yes	ND
	Autologous	Bone marrow	Yes	ND
		Adipose tissue	Yes	ND
Stromal vascular fraction [72]	Autologous	Adipose tissue	Yes	ND
BMSC [73–75]	Autologous	Bone marrow	Yes	ND
USC [76] and secretome[77]	Autologous or Allogeneic	Urine (kidney)	Yes	ND
iPSC [78]	Autologous	Skin	No	ND
Vascular progenitor cells [79]	Allogeneic	Vessels	Yes	ND
EPC [79, 80]	Allogeneic	Vessels	Yes	ND
Mononuclear cells [81]	Autologous	Bone marrow	Yes	ND

*CT* cells approved or involved in clinical trials, *ND* no data available, *ESC* epidermal stem cells, *MMSC* multipotent mesenchymal stromal cells, *BMSC* bone marrow stem cells, *USC* urine derived stem cells, *iPSC* induced pluripotent stem cells, *EPS* endothelial progenitor cells

Fibroblasts and keratinocytes are common cells used in products for wound and burn healing [41]. Keratinocytes are the major cell component of the epidermis and responsible for its stratified structure and form numerous tight intercellular junctions. Fibroblasts are the main cell type of the dermis and produce ECM components and secrete various growth factors (TGF-β), cytokines (TNF-α), and matrix metalloproteinases, which ensure the ECM formation and keratinocyte proliferation and differentiation [16]. Commercial products such as Epicel, Cryoskin, and BioSeed-S contain keratinocytes; Dermagraft, TransCyte and Hyalograft 3D—fibroblasts; and Apligraf, Theraskin, and OrCell—a combination. The use of these cells enables the large-scale production of standardized product batches. However, these materials are mostly non-permanent bioactive dressings, which provide cytokines, ECM, and growth factors for the successful skin reparation [41–43]. Immune rejection is commonly reported with allogeneic fibroblasts and keratinocytes, [44] but this is mostly shown for allogeneic keratinocytes that can be explained by the difference in HLA expression and cytokine production [45]. Fetal fibroblasts are of particular interest because they can significantly improve skin repair due to the high expansion ability, low immunogenicity, and intense secretion of bioactive substances such as basic fibroblast growth factor, vascular endothelial growth factor, and keratinocyte growth factor. However, ethical issues limit their application [46–49].

Epidermal stem cells (ESC) are of particular interest for skin tissue regeneration as they have favorable features such as high proliferation rate and easy access and keep their potency and differentiation potential for long periods [65, 82]. They are one of the skin stem cell types, either heterogeneous or autogenous origins (Table 2). ESC are mostly connected to the process of skin regeneration [17]. They are rare, infrequently divide and generate short-lived and rapidly dividing cells, which are involved in the regeneration process [65]. Their main population, responsible for skin repair, is located in the basal layer of the epidermis; however, they can also be revealed in the base of sebaceous glands and the bulge region of hair follicles [6, 65, 82]. However, while working with ESC culture, we may face progressive aneuploidy or polyploidy and mutation accumulation after several passages. Moreover, as they can be easily derived from the patient’s skin and transplanted to the same patient, ESC are not restricted by ethical issues. Grafts containing autologous holoclones ESC have proven to be effective in treating vast skin defects: epidermolysis, skin and ocular burns, etc. [83, 84].

**Table 2:** Subtypes of skin stem cells

Cell type (Refs)	Localization	Specific markers
Epidermal stem cells [63, 64, 85, 86]	Basal layer of the epidermis	b1^high^/melanoma chondroitin sulfate proteoglycan positive, α6^high^/CD71^dim^, p63
Melanocyte stem cells [87–89]	Follicle bulge region and hair germ	Dct, Pax3, Sox
Follicular stem cells [90–93]	Follicle bulge region	CD34, CD200, K15, K19, Lgr5, Lhx2, NFATC1, NFIB, PHLDA1, Sox9
Hematopoietic stem cells [94]	Follicle dermal papillae	CD34 for lymphoid and hematopoietic progenitor cells
Sebaceous gland stem cells [95]	Sebaceous glands and infundibulum	Blimp1
Mesenchymal stem-like cells[96]	Dermis	CD70+, CD90+, CD105+, CD34-
Neural progenitor cells [97]	Follicle dermal papillae	S100 for schwannomas, peripheral neural tissue astrocytes; HMB45, a neuraminidase-sensitive oligosaccharide side chain of a glycoconjugate

*Refs* references

Mesenchymal stromal cells (MSC) have similar (not identical) features as ESC and can be derived from various tissues, even the skin as mentioned previously [98]. They have a high differentiation potential and a certain degree of plasticity and may generate cells of mesodermal, ectodermal, and endodermal lineages [99]. Moreover, paracrine, trophic, and immunomodulatory MSC properties enable their clinical use [100, 101]. MSC can migrate to the injured tissues, differentiate, and regulate the tissue regeneration by the production of growth factors, cytokines, and chemokines [102]. Their immunomodulatory activity is based on the release of anti-inflammatory cytokines and the inhibition of proliferation of CD4^+^ and CD8^+^ natural killer cells, T cells, and B cells. MSC are considered to be hypoimmunogenic because they do not express class I and II molecules of the major histocompatibility complex (MHC) and co-stimulatory proteins (e.g., CD40, CD80, CD86). Therefore, the transplantation of allogenic MSC has a low risk of the immune rejection [103–105]. In burn therapy, adipose-derived stromal cells refined from the stromal vascular fraction are widely applied because of their easy access and isolation procedure and inspiring improvement of the healing processes [106–108]. They are showed to preserve their therapeutic effects after freezing that ensures their multiple use [109]. It is worth mentioning that even the freshly isolated stromal vascular fraction is showed to be effective in burn therapy [110], but compared to adipose-derived stromal cells, it can release high concentrations of inflammatory mediators [111]. However, the number of randomized controlled preclinical and clinical trials remains insufficient [106].

Among the MSC derived from other tissues (adipose tissue, umbilical cord, etc.) the MSC derived from bone marrow (BMSC) requires special attention. They also possess plasticity and can differentiate into tissues of mesodermal, ectodermal, and endodermal origin [112, 113]. BMSC are considered to participate in the skin development. It has been reported that bone marrow can generate not only hematopoietic and mesenchymal cells but also fibroblast-like cells that are located in the dermis and actively proliferate in the skin during the regeneration processes [69, 114, 115]. The possible disadvantages of BMSC are that the tumor microenvironment may induce changes in the angiogenesis ability and anti-tumor response. Moreover, they may generate tumor-associated fibroblasts and shift a normal immune cell phenotype to an immunosuppressive and tumor promoting one [116].

However, nowadays, the greatest interest in tissue regeneration belongs to induced pluripotent stem cells (iPSC); using somatic cell reprogramming like a magic wand, we can develop patient-specific cells with a tailored phenotype and apply them in clinics [117]. The most commonly used cells for cell reprogramming are dermal fibroblasts, melanocytes, and keratinocytes since they can be easily accessed and isolated from punch biopsies [118]. Research has shown that both murine and human iPSC can be differentiated into dermal fibroblasts [119], keratinocytes [120], and melanocytes [121], opening a door for iPSC technology into dermatology applications. The interesting fact is that fibroblasts achieved via this technique may show increased properties compared to those of the parental fibroblasts, e.g., the exceeded ECM production [122]. This might be related to the changed epigenetic signature that occurs during iPSC differentiation and is critical for their use in skin tissue regeneration. However, when cells are reprogrammed with tumorigenic c-Myc and this transgene remains in iPSC, the risk of tumor formation increases, because c-Myc might be reactivated [123]. Since modern methods for cell purification cannot ensure the full separation of differentiated cells from iPSC, undifferentiated and partly differentiated cells may be implanted into a patient and increase the possibility of tumor formation [124].

## Growth factor therapy

Growth factor therapy is to administrate pro-epidermal growth factors to promote wound healing. These growth factors are bioactive molecules secreted by the body whose function is to stimulate the growth and propagation of cells involved in skin wound healing and inflammation. The use of extra-growth factor increases the number of wound-healing cells, causing faster wound healing. Despite their variety, there are five types commonly used as invigorating molecules in wound healing and regaining via benign tissue repair processes (Table 3). They include compounds influencing epidermal tissue regrowth (epidermal growth factor (EGF); hepatocyte growth factor (HGF)), anti-scarring (transforming growth factor (TGF-ß3)), pro-angiogenesis (vascular endothelial growth factor (VEFG); platelet-derived growth factor (PDGF)), and stromal cell growth (fibroblast growth factor (FGF)). A combination of multiple growth factors may efficiently improve cellular functions: proliferation, migration, differentiation, collagen remodeling, inhibition of fibroblast overgrowth, ECM deposition, etc. Therefore, strategies to control growth factors release may prompt skin tissue regeneration. To optimize substance delivery and loading, bioactivity, therapeutic functionality, dosage form stability, etc., it is vital to develop platforms such as hydrogels, microbeads, or tissue-engineered constructs.

**Table 3:** Growth factor therapy for skin tissue repair

GFs (Refs)	Delivery approach	Dose	In vivo experiment	Outcomes
EGF [125, 126]	Topically HA-EGF conjugate immobilized within HA films	1 μg per patch once	SD rat (full-thickness dorsal skin excision)	- Being secreted by the platelets and macrophages; - Stimulating proliferation of fibroblasts, the cells that produce collagen; - Reducing the healing time of wounds when applied topically
	Topically rhEGF-loaded lipid nanoparticles	20 μg per scar tissue twice a week	White pig (full-thickness dorsal skin excision)	
KGF [127]	Topically KGF covalently attached to a fluorescent matrix-binding peptide encapsulated within fibrin	500 ng/ml	Athymic mouse (full-thickness dorsal skin excision)	- Promotes keratinocytes growth
TGF-β1 [128]	Topically Incorporated into polyoxamer gel	1 μg per wound	SD rat (full-thickness skin excision)	- Stimulating growth and migration of keratinocytes and fibroblasts to the affected area - Promoting the growth of new blood vessels (angiogenesis), ensuring adequate blood supply to the healing wound
TGF-β2 [129]	Subcutaneous implantation Gelatin microspheres	0.5 μg per implant	Athymic rat (subcutaneous implantation)	
HGF [130]	Subcutaneous injection	2 mg per scar tissue once	Rabbit (full-thickness skin excision)	- Reducing scarring
VEGF [131]	Implantation VEGF-loaded alginate microspheres	2 and 4 μg	Wistar rat (small incision in the groin)	- Enabling the most extensive blood vessel formation with microspheres containing 4 μg of VEGF
PDGF Regranex® [132]	Topically Carboxymethyl-cellulose hydrogel	100 μg/g	Patients with type 1 or type 2 diabetes suffering from chronic ulcers	- Being secreted by the platelets, - Attracting fibroblasts and macrophages to the area of injured tissue
TGF-β3 [133]	Topically BMSC overexpressing TGF-β3	0.5 ml (1.3 × 10^5^ cells/ml)	Rabbit (full-thickness skin excision)	- Reducing scar depth and density
bFGF [134]	Topically Poly(ethylene glycol)-poly(dl-lactide) microfibrous mats containing bFGF	ND	Diabetic SD rat (full-thickness dorsal skin excision)	- Enabling higher complete wound closure rate - Stimulating collagen deposition and ECM remodeling bFGF-loaded mats
HGF+bFGF [135]	Topically Collagen/gelatin sponge	10 μg/cm^2^ + 7 μg/cm^2^	C57BL/6JJcl mouse (full-thickness dorsal skin excision)	- Dual release of HGFC and bFGF ensured re-epithelization and angiogenesis.
Platelet-rich fibrin extract [136]	Topically Gelatin gel	3.3 ml of blood per defect	Wistar rat (full-thickness dorsal skin excision)	- Promoting neovascularization and formation of granulation tissue. - Epidermalization started in 1 week
VEGF+PDGF+ bFGF+EGF [137]	Topically Collagen-HA membrane	0.1 μg/mg (each)	Diabetic SD rat (full-thickness dorsal skin excision)	- Increasing wound healing rate - Enhancing the collagen deposition and maturation of vessels.

*GFs* growth factors, *EGF* epidermal growth factor, *KGF* keratynocyte growth factor, *HGF* hepatocyte growth factor, *VEGF* vascular endothelial growth factor, *PDGF* platelet-derived growth factor, *TGF* transforming growth factor, *FGF* fibroblast growth factor, *bFGF* basic FGF, *HA* hyaluronic acid, *ND* no data available, *Refs* references, *wk* week

To improve re-epithelialization after a burn injury, growth factors such as EGF and HGF are applied. EGF and HGF are shown to enhance epithelial cell proliferation, growth, and migration. Their potential in skin tissue regeneration is intensively studied, and various approaches to deliver them are under investigation (Table 3). For example, Lee at al. [138] achieved the improved wound healing of laser-induced burn after treatment with recombinant EGF conjugated with low molecular weight protamine. Regarding HGF, there are few in vivo studies [130, 139, 140]. The EGF efficacy was proven in clinical trials [141, 142].

Angiogenesis in a defect site can be promoted by PDGF and VEGF. PDGF-BB is approved by FDA for diabetic ulcer treatment [143], but it has low success in clinics probably due to its damage by proteolytic enzymes or low expression of PDGF-receptors. VEGF showed high efficacy in experiments in vivo (e.g., [144]) and passed a phase I trial proving its safety and efficacy in treatment of chronic wounds [145]. To promote vessel formation, both PDGF and VEGF require constant application during a treatment period that has induced research to develop delivery systems with sustained release. For instance, Tan et al. [146] revealed VEGF-loaded collagen scaffolds significantly improved the wound healing processes in diabetic rats followed by the increase in VEGF level in tissue and induced angiogenesis. Moreover, Gorkun et al. [147] showed that VEGF-induced spheroids from adipose-derived stromal cells encapsulated within modified fibrin gel can form tubule-like network that might be interesting as a new approach to enhance angiogenesis in a wound and improve skin tissue regeneration.

The increased stromal cell growth can be achieved by the application of FGF. FGF-2 (bFGF) was shown to control ECM formation, and its use enabled the decreased scar formation and inhibition of TGF-β1/SMAD-dependent pathway [148]. Treating deep partial-thickness burns in humans, Ma et al. revealed that recombinant aFGF accelerated the healing rate and the healing process required less time.

The main anti-scarring agent is TGF-ß3. In TGF family, TGF-β1 and TGF-β2 stimulate fibroblast differentiation, contraction, ECM synthesis and deposition, and scarring and TGF-β3 enables the reduction in scar formation. The concentration of TGF-β isoforms varies in the fetal and adult wound healing process; in the first, the TGF- β 3 concentration is high, but TGF-β1 and TGF-β2 isoforms are absent or in a small amount, while in the second, the situation is opposite and the high TGF-β1 and TGF-β2 concentrations are caused by the platelet degranulation and synthesis in monocytes during inflammation. When TGF-β1 and TGF-β2 isoforms were blocked and TGF-β3 isoform was externally added, the wound healing occurred with the less remarkable scar formation than that in control. However, blocking all three isoforms did not ensure the scarring decrease that the complexity of molecular pathways shows [149–151]. Clinical trials showed that avotermin (TGF-β3) ensured scar reduction and was well tolerated [152–154].

Growth factors for skin wounds are often applied locally (topically). One advantage of growth factor therapy is that it uses the body’s own cells to promote healing. Its use may also speed up the time it takes for wounds to heal, resulting in a greater reduction of disability or discomfort for the patient. Various delivery systems are offered to ensure growth factor stability and controlled release in wounds: particulate systems, scaffolds, hydrogels, and their combinations (described in [155]). Moreover, devices such as microneedles [156] and jet injectors [157] are of potential interest although to date no studies where they have been applied to treat burns were found.

Since it is often applied topically, the incidence of systemic side effects is minimal. However, for example, high VEGF serum level causes anasacra, edema, and edema-associated burn complications although, in general, VEGF is considered to promote burn healing [158]. EGF and PDGF can lead to the hypertrophic scarring [159]. Also, theoretically, growth factor therapy of wounds may induce oncogenesis (for instance, TGF-β can be both a pro-oncogenic and tumor suppressing factor [160], and VEGF is involved in tumor formation [161]), but in pre-clinical and clinical trials, tumor development was not revealed [162, 163]. Further long-term trials are required to confirm and strengthen growth factors safety.

In some cases, the use of a single growth factor may be insufficient because of the complexity of molecular pathways and wound chronicity that reveals a need to develop multiple growth factor systems with sustained release. For example, Lai et al. [137] designed a collagen-HA membrane with immobilized VEGF, PDGF, bFGF, and EGF and showed that it efficiently induced the increase in wound healing rate by enhancing collagen deposition and neovasculogenesis compared to the control group.

## Scaffold for skin wound healing

Biomaterials are a crucial part of the different dressings and tissue-engineered constructs (Table 4) used in burn therapy. The main idea in using them is to imitate the skin ECM formed by collagen, elastin, proteoglycans, nidogen, laminin, and perlecan [20, 21] and its properties: the skin’s strength is enabled by collagen, elastin ensures its elasticity and flexibility, and proteoglycans provide hydration and viscosity [20]. In skin grafts and substitutes, biomaterials of various origins (natural, synthetic, or semi-synthetic) are used and their choice in the scaffold fabrication is essential because this can influence the in situ regeneration, with their features regulating cell behavior and enabling new tissue formation. The main requirements are biodegradability, temporary mechanical support, and permeability. Depending on the approach, scaffolds may be with or without cells, and the latter can be divided into dermal, epidermal, and epidermal-dermal composites [41].

**Table 4:** Scaffolds applied in the skin tissue regeneration and wound healing

Scaffolds (Refs)	Origin	BD	Cell component	CA	Example of commercial products
Decellularized material-based					
Small intestine, acellular lyophilized [164]	Porcine	Yes	Not included	Yes	OASIS Wound Matrix
Dermis, acellular lyophilized [165, 166]	Allogeneic	Yes	Not included	Yes	AlloDerm, Karoderm, SureDerm
Dermis, acellular pre-meshed [167]	Allogeneic	Yes	Not included	Yes	GraftJacket
Dermis, acellular lyophilized, coated with elastin hydrolysate [166]	Bovine	Yes	Not included	Yes	Matriderm
Dermis, acellular diisocyanate cross-linked [168, 169]	Porcine	Yes	Not included	Yes	Permacol Surgical Implant
Collagen-based scaffolds					
Collagen [50, 58, 60]	Bovine	Yes	Allogeneic keratinocytes and fibroblasts	Yes	Apligraft
			Autologous keratinocytes and fibroblasts	Yes	PermaDerm
Collagen, aldehyde cross-linked reconstituted [170]	Porcine	Yes	Not included	Yes	EZ Derm
Collagen, sponge [52]	Bovine	Yes	Allogeneic keratinocytes and fibroblasts	Yes	OrCel
Collagen, cross-linked Glycosaminoglycan Polysiloxane [171]	Bovine/synthetic	Yes/no	Not included	Yes	Integra Dermal Regeneration
Collagen, cross-linked Glycosaminoglycan Polysiloxane [70]	Bovine/synthetic	Yes/no	Autologous adipose-derived regenerative cells	No	ND
Collagen, lyophilized cross-linked sponge, heat-denatured Silicone [172, 173]	Bovine/synthetic	Yes/no	Not included	Yes	Terudermis
Atelocollagen Silicone/silicone fortified with silicone gaze TREX [172, 174]	Porcine/synthetic	Yes/no	Not included	Yes	Pelnac Standard/Pelnac Fortified
Collagen Silicone, film Nylon, mesh [53, 175]	Porcine/synthetic	Yes/no	Allogeneic fibroblasts	Yes	Biobrane/Biobrane-L, TransCyte
Hyaluronic acid-based					
Hyaluronic acid membrane (microperforated) [56, 57, 176]	Recombinant	Yes	Autologous keratinocytes and fibroblasts	Yes	TissueTech Autograft System, LaserSkin (Vivoderm)
	Allogeneic	Yes	Autologous fibroblasts	Yes	Hyalograft 3D
HYAFF, derivative of hyaluronan Silicone, membrane [177]	Allogeneic/synthetic	Yes/no	Not included	Yes	Hyalomatrix PA
Other biopolymer-based					
Silk fibroin/alginate, sponge [178]	Xenogeneic/synthetic	Yes/no	Not included	No	ND
Cellulose, nanofibrils [179, 180]	Recombinant	No	Not included	No	ND
Synthetic material-based					
Polyethylene oxide terephthalate/Polybutylene terephthalate [55]	Synthetic	No	Autologous keratinocytes and fibroblasts	Yes	PolyActive
Polyglycolic acid/polylactic acid Extracellular matrix, derived from fibroblasts [54, 181]	Synthetic	Yes	Allogeneic fibroblasts	Yes	Dermagraft

*BD* biodegradability, *CA* commercial availability, *ND* no data available

To date, most products available on the world market contain collagen or decellularized tissues (Table 4). This is not surprising because one of the main skin component is collagen types I and III, and therefore, the product design will be more similar to the native tissue than others [139]. However, collagen possesses poor mechanical properties, and most scientists and manufacturers try to improve them via cross-linking or reinforcing with synthetic materials such as polylactide, polycaprolactone, and their copolymers [182, 183]. For instance, in TranCyte, the collagen gel is fortified with nylon mesh and covered with a silicone film; the latter enables the maintenance of moist environment. The products based on decellularized materials have more clinical limitations than collagen-based scaffolds mainly because they require specific raw materials (especially, allogeneic) and can evoke the strong immune response and calcification (especially, xenogeneic). What is remarkable is that the first FDA-approved skin substitute, Apligraf, contains bovine collagen [87].

It is worth mentioning that hydrogel has proven to provide the most favorable conditions for the burn healing process and is widely applied in tissue engineering [184]. The abovementioned collagen is also a gel, and apart from collagen, gels such as fibrin, hyaluronic acid, chitosan, and alginate are used in the skin substitute and bio-ink production (Table 5). The structure and properties of hydrogels (3D network, hydrophilicity, etc.) can be easily modified and are similar to those of the native ECM, enabling not only cell proliferation and differentiation but also in situ cell recruitment. Gels provide adequate moist environment that is favorable for the burn healing process [185]. Moreover, they can deposit and deliver bioactive compounds, which then enhance the healing process [186–190].

**Table 5:** Hydrogels for cell and growth factor delivery in the skin tissue regeneration

Polymer type	Hydrogels (Refs)	Origin	BD	CA	FDA approved	Commercial product
Protein	Collagen [58]	Xenogeneic	Yes	Yes	Yes	Apligraf
	Gelatin [191, 192]	Xenogeneic	Yes	Yes	No	ND
	Fibrin [193]	Allogeneic	Yes	Yes	No	AcuDress
Polysaccharide	Chitosan [194]	Xenogeneic	No	Yes	No	ND
	Hyaluronic acid [176]	Recombinant, allogeneic	Yes	Yes	Yes	LaserSkin
	Dextran [195, 196]	Xenogeneic (microbial)	Yes	Yes	No	ND
	Alginate [197]	Xenogeneic	No	Yes	Yes	Kaltostat
	Glycosaminoglycan [198]	Xenogeneic, allogeneic	Yes	Yes	No	ND
Polyether	Polyethylene glycol diacrylate [199, 200]	Synthetic	No	Yes	No	ND

*BD* biodegradability, *CA* commercial availability, *ND* no data available, *FDA* Food and Drug Administration

## Delivery approaches

Currently, dressings are the most common form of cell-based products used in burn therapy [41]. Their shape, however, does not provide a possibility to treat large and complex wounds with a heterogeneous surface profile. Therefore, such technologies as cell spraying and three-dimensional (3D) bioprinting were developed for these applications.

3D bioprinting is a multitasking platform that enables in situ cell deposition according to the wound pattern. In 3D bioprinting, cells are distributed within gels, and these mixtures are used as bio-inks. Commonly, the procedure involves printing hydrogel layers, which are further cross-linked via UV, enzymes, ions, etc., to give better support for cells [201–203].

In situ 3D bioprinting was first proposed by Campbell and Weiss [204] for an inkjet bioprinter and is particularly interesting as a delivery approach since it can ensure the full-thickness tissue restoration followed by vasculogenesis due to progenitor cell migration and angiogenesis. Nevertheless, despite promising results, the number of studies, where this technology is used, is limited [205]. This may be caused by the complexity of the equipment and commercial non-availability. For skin tissue applications, there are only two studies performed with human fibroblasts and keratinocytes [205] and amniotic fluid-derived stem cells [206] encapsulated within fibrin-collagen hydrogel and transplanted into a full-thickness wound in nude mice. Thus, after solving technical issues the idea of in situ skin bioprinting could be considered attractive for clinical translation.

Another promising delivery technology is cell spraying that allows clinicians to treat large deep burns [61, 207, 208]. In most studies, scientists used autologous epidermis-derived cells. Cells are not cultured but suspended in saline. The required amount can be derived only from a small donor site [61, 109]. A cell suspension is sprayed homogenously onto a wound so that cells proliferate and improve re-epithelialization [109]. Cell spraying cannot replace common autografting but can be applied easily and early to deep partial thickness burns [208]. Many complications (poor esthetic outcome, hypertrophic scarring, contracture, etc.) may be avoided or decreased due to the early re-epithelialization after a cell spray. Nevertheless, this technology is expensive and needs special equipment, aseptic rooms, and highly qualified personnel as 3D bioprinting.

## Challenges and future directions

To date, despite imperfections, the existing dressings and tissue-engineered skin substitutes have significantly improved clinical insight into burn treatment, allowing clinicians to treat severe cases that increase patients’ survival rates and quality of life [42, 43, 209, 210]. Most of them only aim to temporarily protect the denuded tissue from the aggressive environment and provide cytokines and growth factors to enhance the wound healing process [43]. There is no doubt that commercial products based on autologous cells (fibroblasts and keratinocytes) are close to the native skin and enable the successful skin repair but they cannot fully replace the injured tissue [211].

Many issues limit the introduction and rapid expansion of new products for cell-based therapies. First of all, their production is time- and labor-consuming and requires complex and specific equipment. To cover the extensive burn areas, a huge number of cells is needed, and if they are not autologous or hypoimmunogenic, a substitute can be rejected. These products should be transported and stored under certain conditions, which are hard to maintain, and their shelf-life is short. When autologous cells are applied, the work of cell culture facilities and surgeons should be well coordinated [212]. Moreover, the cost of treatment with skin substitutes is high but the only one function, protective, can be replaced with them [209]: all these tissue-engineered constructs cannot restore thermoregulation, sensation, UV-protection, excretion, perspiration, etc.

Nowadays, in the design of skin substitute, there are three main approaches: cell-based, biomaterial-based, and delivery-based. In the first, scientists try to fabricate skin equivalents using not only fibroblasts and keratinocytes, but also melanocytes and endothelial cells in order to imitate native tissue morphology [213–215]. In many studies, stem cells derived from various sources are used for their properties such as hypo-immunogenicity and high differentiation potential. The use of autologous and allogeneic cells still remains questionable. Although there are studies showing that only autologous cells can promote rapid wound healing [107, 216], a bank of allogeneic cells can provide a possibility to treat quickly patients suffering extensive and deep second-degree burns, and in this case, the most preferable cells are stem cells (e.g., adipose-derived or bone-marrow derived stem cells) possessing hypo-immunogenicity. Moreover, attempts to reproduce skin appendages (for instance, hair follicles and sebaceous glands) in vitro and integrated into skin substitutes [217–219] are made. The second approach tries to functionalize scaffolds with different methods. For example, the immobilization of signaling molecules on their surface can promote the cell proliferation and differentiation and control cell-matrix adhesion [220–223]. According to the third approach, researchers try to develop a new delivery system or to improve the existing ones. To ensure burn healing, cells can be injected intravenously [108], or, more often, they are immobilized on various materials and applied topically as dressings. For sure, dressings are the most common system, but they cannot be precisely adjusted to the wound surface profile. Therefore, technologies such as cell spray and bio-printing are of particular interest and are able to solve this issue [205–208].

**Fig. 1:**
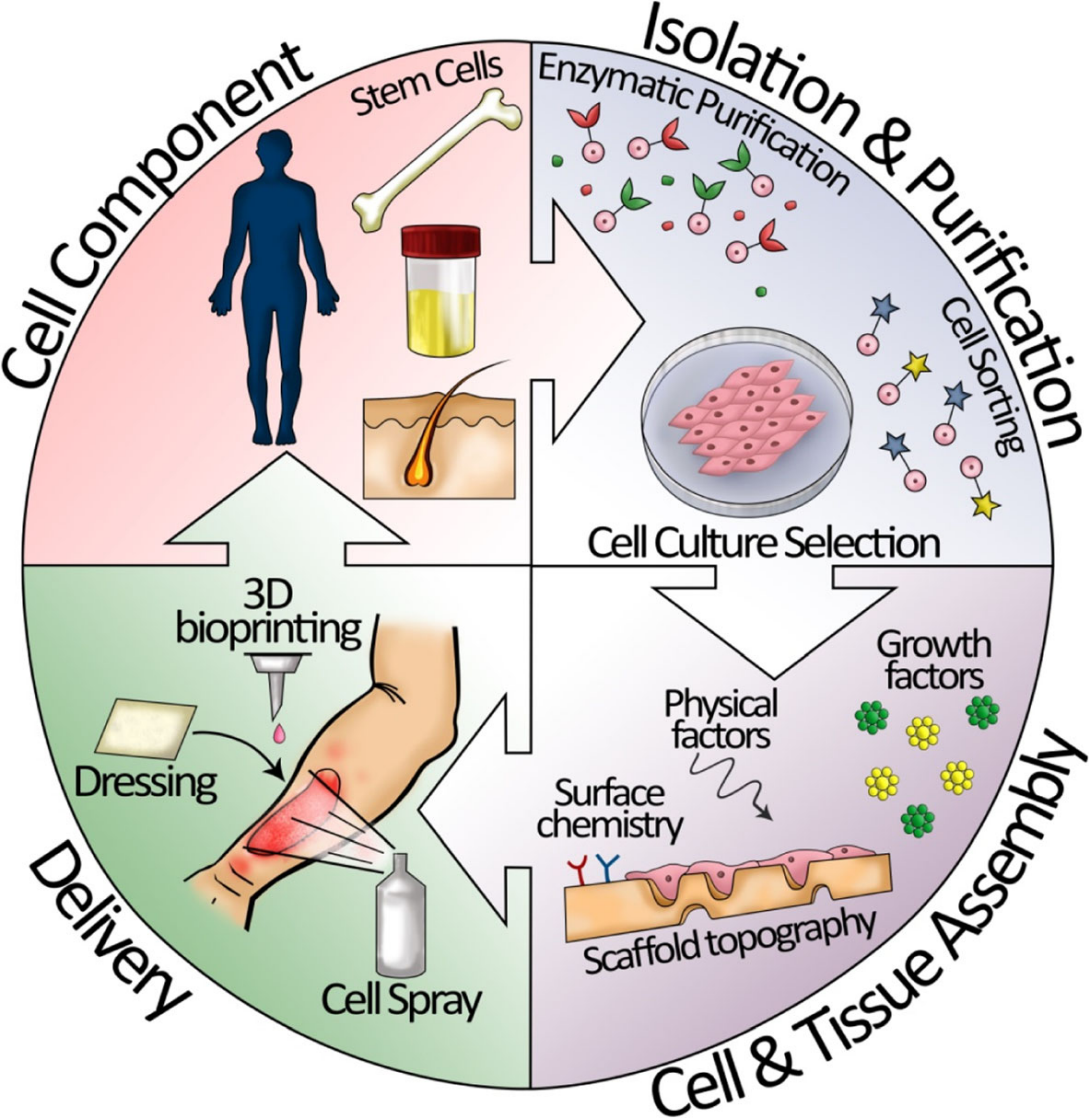
Procedure of autologous stem cell-based therapy on burn injury

Moreover, the stem cells described above can enable true skin regeneration and decrease scar formation and have the clear manipulation step procedure for autologous use (Fig. 1). Preclinical and clinical studies have shown that bone marrow, urine, adipose-derived, and other stem cells can significantly improve the wound healing process in chronic wounds [51, 76, 187, 224, 225]. However, despite these successful results, still FDA has not approved any stem cell-based skin substitute for wound treatment, and for them to make the approval, certain points such as optimal cell type and population and time and way of administration should be clarified. There is an essential need to find out the mechanisms of cell action, survival, and incorporation after transplantation and their stability and differentiation features in the wound microenvironment. Moreover, the delayed postoperative outcomes should be studied in large-scale clinical trials to prove the safety of stem cell-based products. Thus, stem cells are a promising tool for skin substitute design and fabrication for advanced burn therapies.

As the number of findings proving its safety and efficacy is growing, cell-based therapy is becoming a great alternative in burn care. However, some essential points required to standardize all related procedures and prepare guidelines for clinicians are still unclear. In most studies, cells and cell-based products are applied once topically, but these measures can be insufficient in case of extensive burns causing systemic inflammation and hypohydration and only intravenous injections of cells can improve the patient’s condition. Autologous cells are considered to be preferable although their use is impossible in large burns because of the lack of donor sites and time. Moreover, especially in case of acute burns, the successful outcomes of cell-based therapy depend on intervention timing defined by coordination between clinicians and cell facilities staff. Thus, despite outstanding results of cell applications in burn care, the mentioned above issues should be solved to exploit the whole potential of cell-based therapy.

## Abbreviations

3D: Three-dimensional
BD: Biodegradability
bFGF: Basic fibroblast growth factor
BMSC: Bone marrow stem cells
CA: Commercial availability
CT: Clinical trials
ECM: Extracellular matrix
EGF: Epidermal growth factor
EPS: Endothelial progenitor cells
ESC: Epidermal stem cells
FDA: Food and Drug Administration
FGF: Fibroblast growth factor
GFs: Growth factors
HA: Hyaluronic acid
HGF: Hepatocyte growth factor
HLA: Human leukocyte antigen
iPSC: Induced pluripotent stem cells
KGF: Keratinocyte growth factor
MHC: Major histocompatibility complex
MMSC: Multipotent mesenchymal stromal cells
MSC: Mesenchymal stromal cells
ND: No data available
PDGF: Platelet-derived growth factor
Refs: References
TGF: Transforming growth factor
TNF-α: Tumor necrosis factor alpha
USC: Urine-derived stem cells
VEGF: Vascular endothelial growth factor
